# The Relationship Between Parents’ E-Health Literacy and Their Knowledge, Attitudes, and Practices Regarding Pediatric Fever Management: A Cross-Sectional Study

**DOI:** 10.3390/children13101343

**Published:** 2026-10-02

**Authors:** İsa Çelik, Gamzegül Altay, Mehtap Metin Karaaslan

**Affiliations:** Department of Pediatric Nursing, Faculty of Health Sciences, Recep Tayyip Erdogan University, 53100 Rize, Türkiye; gamzegul.altay@erdogan.edu.tr (G.A.); mehtap.karaaslan@erdogan.edu.tr (M.M.K.)

**Keywords:** child, parents, fever, e-health literacy, fever management, pediatric nursing

## Abstract

**Highlights:**

**What are the main findings?**

- Higher parental e-health literacy was positively associated with the total PKAPQ-PFM score, accounting for 10.4% of its variance.
- The least guideline-concordant responses involved cold compresses, alternating antipyretics, complete undressing, touch-based fever assessment, and antipyretic use when the child was not distressed.

**What are the implications of the main findings?**

- Pediatric nurses should integrate e-health literacy support into parent education on childhood fever management.
- These findings may help pediatric nurses prioritize specific misconceptions when designing brief, targeted fever-management education.

**Abstract:**

Aim: This study aimed to describe parents’ knowledge, attitudes, and practices regarding childhood fever management and to examine the association between parental e-health literacy and the total score of the Parental Knowledge, Attitudes, and Practices Questionnaire on Pediatric Fever Management (PKAPQ-PFM). Methods: This descriptive, correlational, cross-sectional study included 442 parents of children aged 0–5 years. Descriptive statistics, simple linear regression, and multivariable linear regression analyses were used for data analysis. Results: Parents’ mean score on the e-Health Literacy Scale was 30.35 ± 6.34, and their mean score on the Parental Knowledge, Attitudes, and Practices Questionnaire on Pediatric Fever Management was 113.17 ± 9.68. Among parents, 49.1% preferred measuring fever from the forehead, and 44.8% considered 38.0 °C and above as the fever threshold for axillary measurement. Paracetamol (81.9%) was the most preferred antipyretic, and 62.7% of households had forehead thermometers. Parents most frequently sought information about the causes of fever (52.5%), dosing intervals for antipyretic medications (37.1%), and the temperature threshold at which medication should be administered (31.9%). E-health literacy was positively and significantly associated with total PKAPQ-PFM scores (R = 0.322, *p* < 0.001) and explained 10.4% of the variance in total PKAPQ-PFM scores (R^2^ = 0.104, *p* < 0.001). This association remained significant after adjustment for sociodemographic characteristics (B = 0.48, β = 0.32, *p* < 0.001). Conclusions: Higher parental e-health literacy was associated with higher total PKAPQ-PFM scores. Strengthening parents’ e-health literacy may be a useful focus for parent education aimed at improving childhood fever management. Practical implications: Pediatric nurses should support parents’ digital health literacy to promote safe fever management practices at home and appropriate medication use.

## 1. Introduction

Fever is a common childhood symptom and represents a physiological host response to infection [1,2]. However, fever continues to be one of the primary sources of anxiety for parents and poses a significant burden on healthcare services [3,4]. Fever is also a frequent reason for pediatric healthcare utilization, including emergency and primary care consultations [5,6].

Parental misconceptions and anxiety related to fever, commonly described as “fever phobia,” remain widespread and may contribute to unnecessary antipyretic use and other non-guideline-concordant practices, including subjective temperature assessment and excessive cooling measures [1,2,7,8,9,10]. Although current guidelines emphasize improving the child’s comfort rather than simply reducing body temperature, parental management often remains focused on fever reduction [11,12].

In today’s digital age, the internet and digital platforms play a central role in parents’ health-related information-seeking behaviors [13]. However, misinformation on the internet may increase parents’ anxiety by making it more difficult to access accurate information. At this point, the concept of “e-health literacy,” defined as the ability of individuals to search for, find, understand, and evaluate health information from electronic sources, gains critical importance [14,15]. Previous studies have examined the relationship between parental e-health literacy and selected outcomes such as anxiety and specific fever-management behaviors [13,14]. Recent studies have also highlighted the importance of parent education and of understanding families’ attitudes, emotions, and caregiving practices in childhood fever management [16,17]. However, evidence remains limited regarding the relationship between parental e-health literacy and parents’ overall knowledge, attitudes, and practices related to pediatric fever management. In the present study, fever management was analyzed more comprehensively across areas such as fever assessment, non-pharmacological management, antipyretic use, and medication safety. In addition, parents’ previous experiences with childhood fever and their fever-related information-seeking behaviors were described to provide a broader context for understanding home fever management.

The aim of this study is to (1) describe parents’ knowledge, attitudes, and practices regarding childhood fever management; (2) examine the association between parental e-health literacy and the total PKAPQ-PFM score, before and after adjustment for sociodemographic characteristics; and (3) describe the information sources parents consult regarding fever management and their levels of trust in these sources.

## 2. Methods

### 2.1. Study Design

This descriptive, correlational, cross-sectional study described parents’ knowledge, attitudes, and practices regarding childhood fever management and examined the association between e-health literacy and the total PKAPQ-PFM score. The research process and reporting followed the Strengthening the Reporting of Observational Studies in Epidemiology (STROBE) guidelines to ensure methodological transparency [18].

### 2.2. Sample

The sample size was calculated a priori using G*Power version 3.1.9.7 (Heinrich Heine University Düsseldorf, Düsseldorf, Germany). The analysis was specified under the F-test family using Linear multiple regression: Fixed model, R^2^ deviation from zero, with one predictor (e-health literacy), which corresponds to a simple linear regression model. Assuming a small effect size according to Cohen’s conventions (f^2^ = 0.02), a significance level of α = 0.05, and 80% statistical power (1 − β = 0.80), the minimum required sample size was 395 participants [19,20]. To account for potential data loss, the target sample size was increased by 10%, to 435 participants. Data collection was completed with 442 parents.

The target population consisted of parents residing in Türkiye who met the study eligibility criteria. Participants were recruited through online platforms using convenience and snowball sampling, without any geographical restrictions. Inclusion criteria included being a mother or father of at least one child aged 0–5 years, having internet access and a digital device (smartphone, tablet, or computer) capable of completing the online survey, having sufficient proficiency in Turkish to read and understand the items in the online data collection form, and voluntarily agreeing to participate in the study by providing digital informed consent. Exclusion criteria included having a child diagnosed with a chronic disease, because disease-specific management, more frequent healthcare contact, and prior medical counseling could influence parental fever-management knowledge and practices. Another exclusion criterion was any cognitive, sensory, or psychological condition in the parent that could hinder reliable completion of the survey items.

### 2.3. Data Collection Tools

#### 2.3.1. Parental Information Form

The researchers developed the form in accordance with the relevant literature [3,9,11,21,22,23,24]. This form consists of three main sections designed to assess participants’ sociodemographic characteristics, experiences related to childhood fever, and fever-related information-seeking behaviors.

Sociodemographic characteristics: The first section includes questions regarding parents’ age, gender, place of residence, education level, employment status, income status, number of children, and the age of the youngest child.

Experiences related to childhood fever: The second section consists of questions examining parents’ practices and experiences, such as the frequency of the child’s fever, healthcare consultations due to fever in the last month, the most frequently used body site for fever measurement, preferred measurement methods/devices, the threshold temperature value accepted for defining fever, and the most frequently preferred type of antipyretic syrup.

Fever-related information-seeking behaviors: The final section covers the information sources parents consult when their child has a fever (healthcare professionals, the internet, social media, etc.), their level of trust in these sources, and the topics on which they most frequently seek information (causes of fever, medication dosage, normal temperature ranges, etc.).

#### 2.3.2. E-Health Literacy Scale (eHEALS)

The e-Health Literacy Scale (eHEALS) was developed by Norman and Skinner [15], and a Turkish validity and reliability study using the scale was conducted by Tamer Gencer [25]. The scale consists of eight items rated on a 5-point Likert-type scale ranging from 1 = Strongly Disagree to 5 = Strongly Agree. Total scores range from 8 to 40, with higher scores indicating higher levels of e-health literacy. The original scale reported a Cronbach’s alpha of 0.88 [15], and the Turkish adaptation reported a Cronbach’s alpha of 0.91 [25]. In the current study, Cronbach’s alpha was 0.94.

#### 2.3.3. Parental Knowledge, Attitudes, and Practices Questionnaire on Pediatric Fever Management (PKAPQ-PFM)

The Parental Knowledge, Attitudes, and Practices Questionnaire on Pediatric Fever Management (PKAPQ-PFM) was developed by the researchers and structured in accordance with current international guidelines and the evidence-based literature [3,9,11,12,21,22,23,24,26,27,28]. The questionnaire was designed to address limitations in existing scales by holistically measuring parents’ knowledge, attitudes, and especially practice skills related to pediatric fever management, such as medication safety, dosing, and storage conditions.

The PKAPQ-PFM was developed to evaluate parents’ knowledge, perceptions, and practices regarding fever management in children; it consists of 30 items covering areas such as the nature of fever and beliefs about fever, the detection and assessment of fever, non-pharmacological fever management practices, and the safe use of antipyretics. All items are rated on a 5-point Likert-type scale (1 = strongly disagree, 5 = strongly agree). Total PKAPQ-PFM scores range from 30 to 150. After reverse-scoring the negatively worded items (items 1, 2, 4, 7, 11, 12, 13, 14, 23, and 28), higher total scores indicate more appropriate levels of parents’ knowledge, attitudes, and practices regarding pediatric fever management. The terms knowledge, attitudes, and practices describe the questionnaire content and do not represent separately validated subscales. Therefore, only the total questionnaire score was used in the analyses.

To evaluate the content validity of the PKAPQ-PFM, the opinions of seven faculty members specialized in pediatric nursing were obtained. The experts evaluated each item’s appropriateness using a 4-point rating scale (1 = not appropriate, 4 = very appropriate). Based on expert evaluations, the item-level Content Validity Index (I-CVI) values ranged from 0.90 to 1.00, and the overall scale-level Content Validity Index (S-CVI) was 0.95. The S-CVI value above the literature threshold of 0.80 indicates that the questionnaire adequately represents the intended construct [29]. The internal consistency of the questionnaire developed for this study was evaluated using Cronbach’s alpha coefficient and was 0.729.

### 2.4. Data Collection Procedure

Research data were collected digitally using Google Forms between December 2023 and March 2024. Participants were recruited using convenience and snowball sampling. The study announcement and survey access link were disseminated through commonly used social media platforms, such as Facebook and Instagram, as well as WhatsApp groups. The survey took approximately 10–15 min to complete. Duplicate submissions were restricted by enabling the “Limit to 1 response” setting in Google Forms. All survey questions were mandatory. A total of 442 survey responses were received. No missing data were present in the variables used for the analyses, and no responses were excluded; therefore, the final analytic sample consisted of 442 parents.

### 2.5. Data Analysis

Statistical analyses were performed using IBM SPSS Statistics software (Version 30.0; IBM Corp., Armonk, NY, USA). Participants’ sociodemographic characteristics and primary health information sources were summarized using descriptive statistics, including frequencies, percentages, means, and standard deviations. The conformity of the data to a normal distribution was assessed by examining skewness and kurtosis coefficients, as well as histograms and Q–Q plots. Based on these analyses, it was determined that the values obtained for the e-Health Literacy Scale (skewness = −0.721; kurtosis = 1.105) and the Parental Knowledge, Attitudes, and Practices Questionnaire on Pediatric Fever Management (skewness = 0.283; kurtosis = −0.286) were within the limits of ±2, confirming that the data met the assumption of normal distribution [30]. Internal consistency was assessed using Cronbach’s alpha [31]. Simple linear regression was first performed to examine the unadjusted association between e-health literacy and the total PKAPQ-PFM score. Multivariable linear regression was additionally performed to examine this association after adjusting for the sociodemographic characteristics (age, sex, place of residence, education, employment status, income, number of children, and age of the youngest child). Categorical variables were dummy-coded. Model assumptions were assessed using Q–Q plots of residuals, residual-versus-fitted plots, variance inflation factors, and the Durbin–Watson statistic. All VIF values were below 1.50, indicating no evidence of problematic multicollinearity. The overall significance of the models was evaluated using the F-test, and the proportion of variance accounted for by each model was reported using the coefficient of determination (R^2^). For all statistical analyses, the significance level was set at *p* < 0.05.

### 2.6. Ethical Considerations

This study was conducted in accordance with the principles of the Declaration of Helsinki. To carry out the research, the necessary scientific and ethical approval was obtained from the University Non-Interventional Clinical Research Ethics Committee (Date: 23.11.2023; Decision No. 2023/258). Prior to data collection, an informed consent form was provided at the beginning of the online data collection form. This form clearly and understandably stated the purpose of the study, the data collection and usage procedures, that participation was entirely voluntary, and that participants could withdraw at any time without providing a reason. The consent form also included the researcher’s contact information for questions about the study. Informed consent was obtained from the participants through a digital approval mechanism on the introductory page of the survey; participants who did not provide consent were not permitted to continue with the survey. To protect participant privacy and anonymity, personally identifiable data, such as names, identification numbers, email addresses, or telephone numbers, were not collected. Access to the collected data was restricted to the research team, and participant privacy was maintained throughout the process by adhering to Google Forms privacy and security protocols. The data obtained were stored in password-protected, secure digital environments accessible only to the research team. Additionally, the necessary permissions for the use of the measurement tools employed in the study were obtained from the relevant authors.

## 3. Results

The mean age of the parents in the study was 32.40 ± 4.69 years, ranging from 20 to 46 years. Most participants were female (88.9%, *n* = 393), university graduates (81.9%, *n* = 362), and lived in the city center (79.8%, *n* = 353). Of the parents, 60.0% (*n* = 265) were employed, and 58.1% (*n* = 257) reported that their income equaled their expenses. Additionally, 61.3% (*n* = 271) of participants had one child, and 40.3% (*n* = 178) had their youngest child aged 3–5 years (Table 1).

Half of the participants (50.0%, *n* = 221) reported that their child had a fever in the last month, while 25.8% of all participants (*n* = 114) reported taking their child to a physician for a high fever during the same period. Healthcare professionals were the most frequently consulted source of information (88.9%, *n* = 393) and were also the most trusted source (96.6%, *n* = 427). The topic parents most frequently sought information on during a fever episode was the causes of fever (52.5%, *n* = 232) (Table 2).

In the study, the mean score on the e-Health Literacy Scale was 30.35 ± 6.34, with an observed range of 8–40, and the mean total score on the PKAPQ-PFM was 113.17 ± 9.68, with an observed range of 92–146. Item-level means are presented in Table 3, and the distribution of item responses is shown in Supplementary Figure S1. The lowest-scoring (least guideline-concordant) items clustered around non-evidence-based practices—cold compresses, antipyretic alternation, complete undressing, palpation-based assessment, and antipyretic use without distress—whereas the highest-scoring items reflected medication-safety behaviors (e.g., checking expiry dates) and supportive care (e.g., encouraging fluids).

Simple linear regression showed that e-health literacy was significantly associated with the total PKAPQ-PFM score, F(1, 440) = 51.05, *p* < 0.001, and accounted for 10.4% of the variance (R^2^ = 0.104). The association was statistically significant (β = 0.32, t = 7.15, *p* < 0.001): each one-point increase in e-health literacy was associated with a 0.49-point increase in the total score (B = 0.49, 95% CI [0.36, 0.63]; Model 1, Table 4). After adjustment for age, sex, place of residence, education, employment status, income, number of children, and age of the youngest child, the association was essentially unchanged (B = 0.48, β = 0.32, t = 6.89, 95% CI [0.34, 0.62], *p* < 0.001); the adjusted model was significant, F(13, 428) = 6.76, *p* < 0.001, R^2^ = 0.170, adjusted R^2^ = 0.145 (Model 2, Table 4). In the adjusted model, male sex (B = −3.15, *p* = 0.029) and high school education (B = −3.50, *p* = 0.005) were the only sociodemographic covariates significantly associated with lower total scores.

## 4. Discussion

The findings showed that e-health literacy was associated with the total PKAPQ-PFM score, and this association remained significant after adjustment for sociodemographic characteristics.

Parents predominantly used forehead-based temperature measurement, particularly infrared thermometers. Guidelines on fever measurement methods recommend axillary measurement with an electronic thermometer, especially for neonates younger than 4 weeks, and either an electronic axillary thermometer or an infrared tympanic thermometer in children aged 4 weeks to 5 years for reliable measurement [7,12,32].

Parents showed variability in the temperature thresholds they considered indicative of fever for axillary measurement. International recommendations indicate that fever thresholds should be interpreted according to the measurement site, the child’s age, and the clinical context [7,13,32]. Misunderstanding these thresholds may contribute to unnecessary concern and to premature use of antipyretics aimed primarily at reducing body temperature [22].

Paracetamol was the most commonly preferred antipyretic among parents. International clinical practice guidelines [11,22,26] emphasize that the primary aim of antipyretic use is not to reduce body temperature per se, but rather to improve the child’s comfort and reduce illness-related distress. In contrast, the widely prevalent phenomenon of “fever phobia” among parents may lead to the unnecessary use of antipyretics beyond guideline recommendations, often with the sole aim of lowering body temperature [1,3]. In this context, ensuring that parents’ decisions about medication use in fever management are shaped by evidence-based information is critical to preventing unnecessary or inappropriate antipyretic use. Visual, evidence-based education during discharge or outpatient follow-up visits increases parents’ knowledge of correct antipyretic dosing and indications [16,33].

Parents commonly reported having more than one type of thermometer at home, particularly forehead and digital axillary devices. Previous research indicates that parents may worry about correct temperature-measurement technique [34], highlighting the importance of clear guidance on appropriate thermometer use [16,17].

In the PKAPQ-PFM, the five items with the lowest scores were, respectively, “I apply cold compresses to the neck, armpits, and forehead when my child has a fever,” “I alternate antipyretics (e.g., by using medications with different active ingredients in turn),” “I remove all of my child’s clothing when they have a fever,” “I determine if my child has a fever by touching their skin or forehead and administer antipyretics if necessary,” and “I do not give antipyretics if my child has a fever but does not seem uncomfortable or irritable.” The last item reflects guideline-concordant practice; its relatively low score indicates lower agreement with withholding antipyretics when the child is not distressed. These findings suggest that parents may still rely on non-evidence-based practices in pediatric fever management, including external cooling, alternating antipyretics, excessive undressing, fever assessment by touch, and antipyretic use even when the child appears comfortable. International clinical practice guidelines emphasize that external cooling methods, such as cold or lukewarm compresses or bathing, provide only a short-term reduction in body temperature and may reduce the child’s comfort by causing peripheral vasoconstriction, shivering, and increased metabolic stress [11,12,32]. Similarly, guidelines do not recommend the routine alternating or combined use of antipyretics, as these practices do not provide additional clinical benefit and may increase parental confusion, dosing errors, toxicity, and acute kidney injury risk [11,12,22,35]. Nevertheless, alternating antipyretic use remains common among parents (42.4–49%), which may be related to fever phobia, unreliable information sources, and inadequate health literacy [1,7,21,35,36].

The tendency to completely undress febrile children also conflicts with current recommendations, which emphasize light, comfortable clothing appropriate to the ambient temperature rather than aggressive attempts to reduce body temperature [9,12,21]. Such practices may increase shivering, metabolic rate, and physiological stress, and may be associated with fever phobia, inadequate health literacy, and reliance on unreliable information sources [32,37,38]. In addition, although many parents still assess fever by touching the child’s skin or forehead, clinical guidelines recommend basing antipyretic decisions on objective temperature measurements with age-appropriate instruments and the child’s clinical condition rather than subjective assessment [2,12,14,21,26,39,40]. Finally, antipyretics should primarily be used to improve the child’s comfort, not merely to normalize body temperature; therefore, routine use is not recommended when the child has no significant distress [22,26,36]. Recent evidence shows substantial heterogeneity in how the pediatric literature defines and assesses fever-related discomfort, underscoring the need for a standardized definition and assessment framework [41]. More recently, an international Delphi consensus proposed an operational definition and a pragmatic clinical framework for assessing fever-related discomfort in children [42]. Fever phobia also remains a persistent concern among caregivers and may contribute to unnecessary or non-guideline-concordant fever-management practices [43]. These findings highlight the need for structured, evidence-based education by pediatric nurses to reduce fever phobia, improve parental health literacy, and prevent unnecessary or inappropriate fever management practices [1,2,3,14].

In the unadjusted model, e-health literacy accounted for 10.4% of the variance in the total PKAPQ-PFM scores. After adjustment for sociodemographic characteristics, e-health literacy remained positively associated with total PKAPQ-PFM scores, while the overall model accounted for 17.0% of the variance. The e-health literacy coefficient changed only slightly between the unadjusted and adjusted models, indicating that the observed association was relatively stable after accounting for the measured sociodemographic characteristics. These findings are consistent with the possibility that parents with higher e-health literacy may be better able to access, evaluate, and apply reliable information related to fever assessment and management [2,13,14]. This association may reflect a greater capacity among parents with higher e-health literacy to critically appraise digital health information and translate reliable information into guideline-concordant fever-management practices at home [11,12,22]. The adjusted analysis also showed lower total PKAPQ-PFM scores among male parents and those with high school education compared with the reference groups, suggesting that some sociodemographic characteristics may also be associated with fever-management knowledge and practices. These findings should be interpreted in light of the sample composition, which predominantly included mothers, university-educated parents, urban residents, and digitally connected participants. Evidence-based educational materials and distance education applications delivered through digital platforms may facilitate parents’ access to accurate health information and support the development of safe fever management behaviors [16].

Parents appeared to actively seek information during fever management. Most parents reported consulting healthcare professionals as their primary source of information (88.9%) and identified them as the most trusted source (96.6%), whereas relatives or friends and websites were used less frequently and perceived as less reliable. This finding aligns with previous studies showing that healthcare professionals are the primary and most trusted source of information for parents [2,7]. In addition, a recent systematic review found that, over time, both doctors and the internet have increasingly been used as first-line sources of information by caregivers managing childhood fever [44]. Parents most often sought information about the causes of fever, antipyretic dosing intervals, and the temperature threshold for medication use, indicating a need for practical, reliable guidance on home fever management and for easily accessible, trustworthy digital resources, particularly when and how to use antipyretics safely [3,40,44].

## 5. Implications for Nursing

The findings suggest that pediatric nurses may consider incorporating e-health literacy support into parent education on fever management. In clinical encounters, nurses should assess not only parents’ knowledge of fever management but also their digital health-seeking behaviors and ability to evaluate online health information. Parents with limited e-health literacy may benefit from additional education and counseling, particularly in identifying reliable, evidence-based health information.

Nurses should promote a family-centered approach that considers the child’s overall clinical condition and comfort rather than focusing solely on fever level. Nurse-led web-based education programs and digital information tools may improve parents’ access to accurate information, support safe home-based fever management, reduce unnecessary healthcare utilization, and strengthen parental decision-making.

However, these implications should be interpreted cautiously when considering parents with lower levels of digital access or health literacy, who were underrepresented in the present sample.

## 6. Limitations

This study has several limitations. Since data were collected when children were stable, parents’ responses may not fully reflect their actual behaviors during a real fever episode. The cross-sectional design does not allow causal interpretation of the association between e-health literacy and the total PKAPQ-PFM score. Because convenience and snowball sampling were used and the number of individuals who received or viewed the survey invitation could not be determined, a response rate could not be calculated. This recruitment approach may have introduced self-selection and nonresponse bias, as parents who were more interested in fever management, more frequent users of digital health information, or more health-conscious may have been more willing to participate. Consequently, the descriptive findings may not fully represent e-health literacy levels and fever-management knowledge, attitudes, and practices in the broader parent population, and the selective composition of the sample may also have affected the magnitude of the observed association. The sample predominantly consisted of mothers, university-educated parents, urban residents, and individuals with digital access, which may limit the generalizability of the findings to parents with different sociodemographic and digital-access profiles. The self-reported nature of the data may also have led to recall bias or socially desirable responses. Finally, although e-health literacy was significantly associated with the total PKAPQ-PFM score, it accounted for only 10.4% of the variance, indicating limited explanatory power.

A further limitation concerns the measurement tool: the PKAPQ-PFM was newly developed for this study, and no pilot testing was conducted before the main data collection. Its psychometric evaluation was limited to content validity and internal consistency (Cronbach’s α = 0.729). Importantly, the authors did not establish construct validity through factor analysis, a key limitation of the current psychometric evaluation. The relatively modest reliability indicates that further psychometric validation is needed before recommending the tool for wider use.

## 7. Conclusions

This study showed that parents’ e-health literacy was significantly associated with the total PKAPQ-PFM score. The findings revealed differences in fever measurement sites, thermometer types, fever threshold values, preferred pharmacological agents, and parents’ information needs. The most frequently sought topics were the causes of fever, antipyretic dosing intervals, and the fever levels at which to administer medication.

Higher e-health literacy was associated with higher total PKAPQ-PFM scores. However, some parents continued to use non-evidence-based practices, including cold applications, alternating antipyretics, excessive undressing, touch-based fever assessment, and antipyretic use without significant distress. These findings highlight the need for structured, evidence-based nurse-led education to support safe home-based fever management and align parental practices with clinical guidelines.

## Figures and Tables

**Table 1:** The demographic characteristics of the study participants (*n* = 442).

Variables	Mean ± SD	Min–Max
Age (years)	32.40 ± 4.69	20–46
	*n*	%
Gender		
Female	393	88.9
Male	49	11.1
Education Level		
High School	80	18.1
University	362	81.9
Employment Status		
Employed	265	60.0
Unemployed	177	40.0
Income Status		
Income lower than expenses	60	13.6
Income equal to expenses	257	58.1
Income higher than expenses	125	28.3
Place of Residence		
District	89	20.2
City center	353	79.8
Number of Children		
1	271	61.3
2	137	31.0
3 or more	34	7.7
Age of the youngest child		
0–6 months	77	17.4
7–12 months	70	15.8
1–2 years	117	26.5
3–5 years	178	40.3

Note: SD, standard deviation; *n*, number of participants.

**Table 2:** Experiences related to childhood fever and fever-related information-seeking behaviors (*n* = 442).

Variables		*n*	%
Child had a fever in the last month	Yes	221	50.0
	No	221	50.0
Healthcare consultation due to high fever in the last month	Yes	114	25.8
	No	328	74.2
Frequency of fever episodes	Occasionally	250	56.6
	Very rare	171	38.7
	Very frequent	21	4.8
Body site where fever is measured	Forehead	217	49.1
	Axillary (underarm)	157	35.5
	Ear (tympanic)	54	12.2
	Other (oral, etc.)	14	3.2
Most frequently used measurement method	Infrared thermometer from the forehead	198	44.8
	Digital thermometer from the axilla	139	31.4
	Digital thermometer from the ear	54	12.2
	By touching with the hand	51	11.5
Threshold temperature accepted as fever (Axillary)	38.0 °C and above	198	44.8
	37.4 °C and above	104	23.5
	38.4 °C and above	92	20.8
	37.0 °C and above	48	10.9
Preferred antipyretic medication	Paracetamol	362	81.9
	Ibuprofen	80	18.1
Information sources consulted *	Healthcare professionals	393	88.9
	Relatives or friends	73	16.5
	Websites (blogs, news, etc.)	72	16.3
	Social media (WhatsApp/Telegram groups)	51	11.5
Most trusted information source *	Healthcare professionals	427	96.6
	Relatives or friends	45	10.2
	Websites (blogs, news, etc.)	28	6.3
	Social media (WhatsApp/Telegram groups)	16	3.7
Topics for which information was sought *	Causes of fever	232	52.5
	Medication dosing intervals	164	37.1
	Threshold for medication administration (at what temperature)	141	31.9
	Medication dosage amount	100	22.6
	Normal temperature ranges	92	20.8
	Which medication to administer	85	19.2
	Symptoms of fever	84	19.0
Types of thermometers available at home *	Forehead (temporal)	277	62.7
	Digital (axillary)	270	61.1
	Ear (tympanic)	77	17.4
	Mercury-in-glass	5	1.1

Note: *n*, number of participants. * Total percentage exceeds 100% as participants were allowed to select multiple response options.

**Table 3:** Descriptive statistics for items of the Parental Knowledge, Attitudes, and Practices Questionnaire on Pediatric Fever Management (*n* = 442).

No.	Questionnaire Items	Mean ± SD	Min–Max
1	Having a fever is always dangerous for my child. *	3.24 ± 1.19	1–5
2	A child with a fever should always be examined by a doctor. *	3.15 ± 1.20	1–5
3	The primary goal of antipyretics is to provide comfort to the child rather than treating the underlying cause of the fever.	3.97 ± 0.92	1–5
4	A child with a fever should always receive antibiotic treatment. *	4.46 ± 0.74	1–5
5	Fever is a normal physiological response of the body to fight infections.	4.34 ± 0.67	2–5
6	I can recognize signs of fever in my child (such as loss of appetite, irritability, fatigue, skin flushing, or cold hands and feet).	4.22 ± 0.77	1–5
7	I determine if my child has a fever by touching their skin or forehead and administer antipyretics if necessary. *	2.97 ± 1.27	1–5
8	I determine if my child has a fever by using a thermometer and administer antipyretics if necessary.	4.24 ± 0.69	1–5
9	I know how to use a thermometer correctly to measure my child’s temperature.	4.38 ± 0.61	2–5
10	I do not give antipyretics if my child has a fever but does not seem uncomfortable or irritable.	3.09 ± 1.22	1–5
11	I always give antipyretics if my child has a fever. *	3.46 ± 1.19	1–5
12	I remove all of my child’s clothing when they have a fever. *	2.83 ± 1.16	1–5
13	I apply cold compresses to the neck, armpits, and forehead when my child has a fever. *	2.00 ± 0.91	1–5
14	I give my child a cold shower when they have a fever. *	3.53 ± 1.23	1–5
15	When my child has a fever, I dress them in clothing that is neither too thin nor too thick.	4.16 ± 0.73	2–5
16	I encourage fluid intake when my child has a fever.	4.50 ± 0.55	2–5
17	I feed my child their favorite fruit or vegetable purees when they have a fever.	3.83 ± 0.96	1–5
18	I check the expiration date of the antipyretic medication before use.	4.64 ± 0.55	1–5
19	If the antipyretic has been opened before, I check its odor, color, and whether it has crystallized.	4.45 ± 0.67	1–5
20	If I am opening the antipyretic for the first time, I record the date of opening on the box.	3.80 ± 1.22	1–5
21	I do not use antipyretic medications if more than 6 months have passed since the opening date.	4.25 ± 0.94	1–5
22	I store the antipyretic in its box at room temperature in a cool and dry place.	4.19 ± 0.92	1–5
23	I store the antipyretic medication in the refrigerator after opening. *	3.30 ± 1.35	1–5
24	I read the package insert before using the antipyretic.	4.00 ± 0.94	1–5
25	I know the correct dose (measuring spoon or milliliters) of antipyretic medication for my child.	4.33 ± 0.66	1–5
26	I know the maximum daily dose (total measuring spoon or milliliter amount) of antipyretics I can give to my child.	4.33 ± 0.70	1–5
27	I know how long it usually takes for the fever to subside after administering an antipyretic.	4.02 ± 0.78	1–5
28	I alternate antipyretics (e.g., by using medications with different active ingredients in turn). *	2.35 ± 1.21	1–5
29	I know that antipyretic medications do not prevent febrile seizures (convulsions).	3.18 ± 1.12	1–5
30	I know that the calibration of thermometers can be impaired.	3.97 ± 0.80	1–5

Note: SD = standard deviation. Items marked with an asterisk (*) are reverse-scored, and their means are presented after reverse coding. For all items, higher mean scores indicate more guideline-concordant responses.

**Table 4:** Unadjusted and adjusted associations of e-health literacy with total PKAPQ-PFM scores.

Predictor	B	SE	β	t	*p*	95% CI for B
Model 1. Unadjusted						
Constant	98.240	2.135	—	46.016	<0.001	94.044–102.436
e-Health literacy (eHEALS)	0.492	0.069	0.32	7.145	<0.001	0.357–0.627
Model 2. Adjusted						
Constant	103.260	4.370	—	23.640	<0.001	94.680–111.850
e-Health literacy (eHEALS)	0.480	0.070	0.32	6.890	<0.001	0.340–0.620
Sex (male)	−3.150	1.440	−0.10	−2.180	0.029	−5.990–−0.320
Age (years)	−0.090	0.110	−0.04	−0.830	0.409	−0.310–0.130
Place of residence (district)	−1.390	1.090	−0.06	−1.270	0.206	−3.540–0.770
Education (high school)	−3.500	1.250	−0.14	−2.810	0.005	−5.940–−1.050
Employment status (employed)	0.800	1.010	0.04	0.790	0.430	−1.190–2.800
Income (higher than expenses)	0.130	1.460	0.01	0.090	0.927	−2.740–3.000
Income (equal to expenses)	−0.050	1.310	0.00	−0.040	0.967	−2.630–2.520
Number of children (2)	−1.770	1.000	−0.08	−1.770	0.078	−3.730–0.200
Number of children (3 or more)	−2.320	1.880	−0.06	−1.230	0.218	−6.000–1.370
Age of youngest child (1–2 years)	0.220	1.110	0.01	0.190	0.846	−1.970–2.410
Age of youngest child (0–6 months)	0.550	1.310	0.02	0.420	0.676	−2.030–3.130
Age of youngest child (7–12 months)	−2.330	1.330	−0.09	−1.760	0.080	−4.930–0.280

Model summaries: Model 1: R = 0.322, R^2^ = 0.104, F(1, 440) = 51.050, *p* < 0.001, Durbin–Watson = 1.933. Model 2: R^2^ = 0.170, adjusted R^2^ = 0.145, F(13, 428) = 6.760, *p* < 0.001, Durbin–Watson = 1.980. Note. B = unstandardized coefficient; SE = standard error; β = standardized coefficient; CI = confidence interval. Dependent variable: total PKAPQ-PFM score. PKAPQ-PFM = Parental Knowledge, Attitudes, and Practices Questionnaire on Pediatric Fever Management. Model 1 includes e-health literacy only; Model 2 additionally adjusts for age, sex, place of residence, education, employment status, income, number of children, and age of the youngest child. Reference categories: female (sex), city center (place of residence), university (education), unemployed (employment status), income lower than expenses (income), 1 child (number of children), and 3–5 years (age of the youngest child).

## Data Availability

The data that support the findings of this study are available from the authors upon reasonable request due to privacy.

## Supplementary Materials

The following supporting information can be downloaded at: https://www.mdpi.com/article/10.3390/children13101343/s1, Figure S1: Distribution of responses to each item of the Parental Knowledge, Attitudes, and Practices Questionnaire on Pediatric Fever Management (PKAPQ-PFM), ordered from the highest to the lowest mean score.
