# Supplementary material for: The Relationship Between Parents’ E-Health Literacy and Their Knowledge, Attitudes, and Practices Regarding Pediatric Fever Management: A Cross-Sectional Study
Source: Children (Basel). 2026 Oct 2;13(10):1343. doi: 10.3390/children13101343 (PMC13703384; doi:10.3390/children13101343)
Supplement: Supplementary file 1 [file children-13-01343-s001.zip › children-4549830-supplementary.pdf]

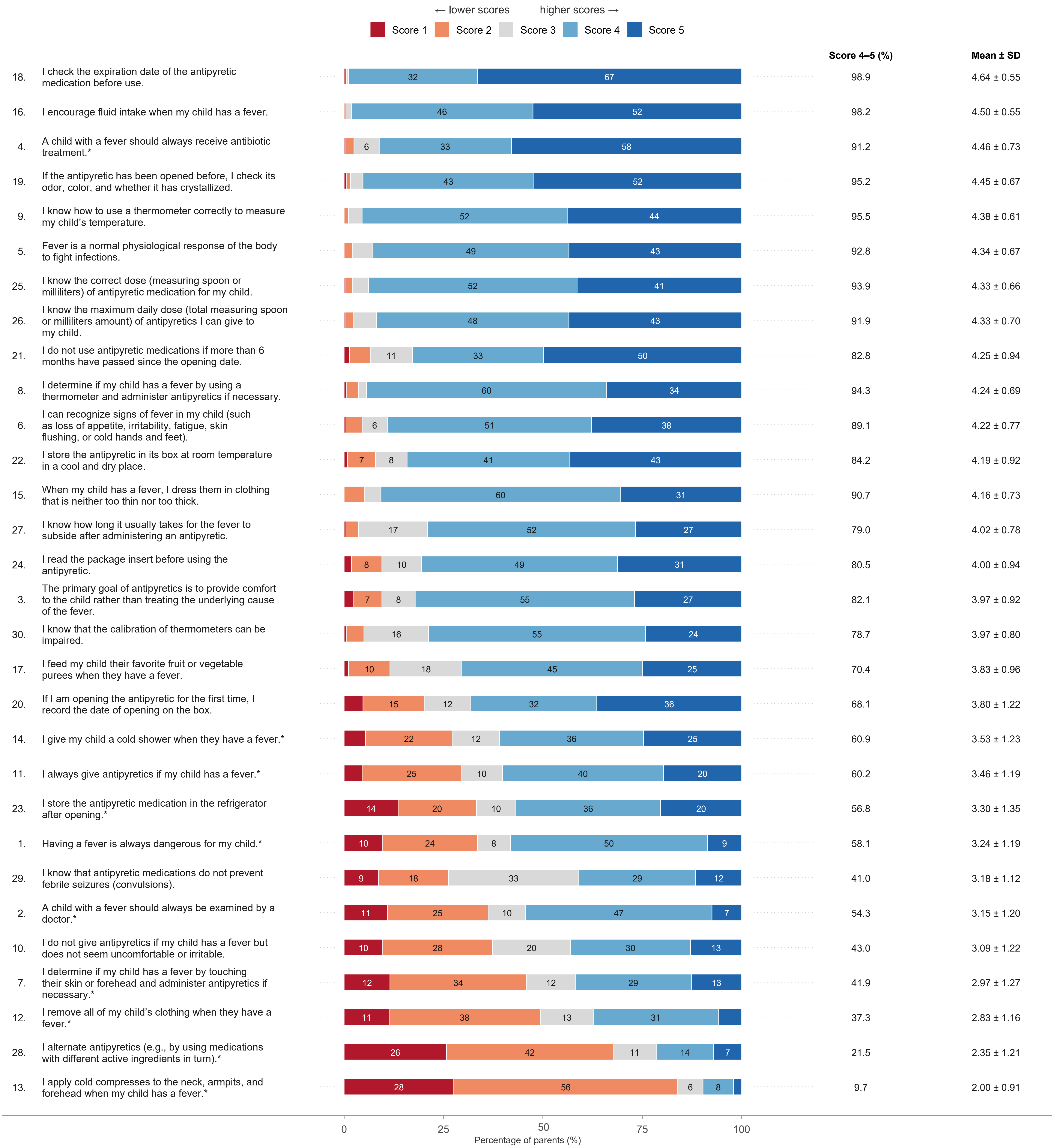

Figure S1. Distribution of responses to each item of the Parental Knowledge, Attitudes, and Practices Questionnaire on Pediatric Fever Management (PKAPQ-PFM), ordered from the highest to the lowest mean score.

Note. n = 442. Items were rated on a 5-point Likert-type scale (1 = strongly disagree to 5 = strongly agree). Scores are item scores after reverse-scoring, so 5 always denotes the most guideline-concordant response and 1 the least. Items marked with an asterisk (*) are reverse-scored, so a low score reflects more frequent endorsement of the stated practice. The questionnaire item number is given before each statement. Score 4-5 (%) = percentage of parents giving a guideline-concordant response (4 or 5 after reverse-scoring). Segment labels are percentages and are shown only for segments of at least 6%; percentages may not sum to 100 because of rounding. SD = standard deviation.
